# Polarized Cdc42 activation promotes polar body protrusion and asymmetric division in mouse oocytes

**DOI:** 10.1016/j.ydbio.2013.01.029

**Published:** 2013-05-01

**Authors:** Benoit Dehapiot, Virginie Carrière, John Carroll, Guillaume Halet

**Affiliations:** a CNRS, UMR 6290, Institut de Génétique et Développement de Rennes, F-35043 Rennes, France; b Université Rennes 1, UEB, IFR 140, Faculté de Médecine, F-35043 Rennes, France; c Department of Cell and Developmental Biology, University College London, Gower street, London WC1E 6BT, United Kingdom

**Keywords:** Oocyte, Mouse, Meiosis, Cdc42, Polarity, Actin

## Abstract

Asymmetric meiotic divisions in mammalian oocytes rely on the eccentric positioning of the spindle and the remodeling of the overlying cortex, resulting in the formation of small polar bodies. The mechanism of this cortical polarization, exemplified by the formation of a thick F-actin cap, is poorly understood. Cdc42 is a major player in cell polarization in many systems; however, the spatio-temporal dynamics of Cdc42 activation during oocyte meiosis, and its contribution to mammalian oocyte polarization, have remained elusive. In this study, we investigated Cdc42 activation (Cdc42–GTP), dynamics and role during mouse oocyte meiotic divisions. We show that Cdc42–GTP accumulates in restricted cortical regions overlying meiotic chromosomes or chromatids, in a Ran–GTP-dependent manner. This polarized activation of Cdc42 is required for the recruitment of N-WASP and the formation of F-actin-rich protrusions during polar body formation. Cdc42 inhibition in MII oocytes resulted in the release of N-WASP into the cytosol, a loss of the polarized F-actin cap, and a failure to protrude the second polar body. Cdc42 inhibition also resulted in central spindle defects in activated MII oocytes. In contrast, emission of the first polar body during oocyte maturation could occur in the absence of a functional Cdc42/N-WASP pathway. Therefore, Cdc42 is a new protagonist in chromatin-induced cortical polarization in mammalian oocytes, with an essential role in meiosis II completion, through the recruitment and activation of N-WASP, downstream of the chromatin-centered Ran–GTP gradient.

## Highlights

► Cdc42–GTP is polarized in the cortex overlying the spindle in mouse oocytes. ► Cdc42 activation is triggered by the Ran–GTP gradient emanating from chromatin. ► Polarized Cdc42–GTP recruits N-WASP to build F-actin-rich cortical protrusions. ► The Ran/Cdc42/N-WASP cascade is essential to second polar body protrusion. ► Cdc42 inhibition also induces central spindle defects in activated oocytes

## Introduction

To become functional gametes competent for fertilization and preimplantation embryonic development, mammalian oocytes execute two rounds of asymmetric meiotic divisions, resulting in the formation of a large oocyte, which retains most of the maternal stores, and two small polar bodies. During the first meiotic division, homologous chromosomes are segregated, and genetic diversity is created through the resolution of the chiasmata (Holt and Jones, 2009). During the second meiotic division, which in mammals is completed only after fertilization, sister chromatids are segregated in a manner that resembles mitotic cell division (Perry and Verlhac, 2008).

The strong asymmetry of oocyte meiotic divisions is driven by spindle positioning in the vicinity of the cortex. During meiosis I (MI), the first meiotic spindle forms in the centre of the oocyte and migrates along its long axis towards the nearest cortical region (Verlhac et al., 2000). Recent studies have revealed the key role of cytoplasmic actin filaments nucleated by Formin-2 (Fmn2) and Spire-type actin nucleators, in driving spindle migration (Azoury et al., 2008; Li et al., 2008; Schuh and Ellenberg, 2008; Pfender et al., 2011). When homologous chromosome separation is initiated, the MI spindle has reached a subcortical location, resulting in a first asymmetric division and the extrusion of one set of chromosomes in the first polar body (PB1). The chromosomes that are retained in the oocyte then promote the assembly of the Metaphase II (MII) spindle, which remains anchored to the cortex of the ovulated oocyte. Upon fertilization, meiosis resumes and a second asymmetric cell division leads to the extrusion of the second polar body (PB2), containing one set of segregated chromatids.

Eccentric spindle positioning, which is instrumental in the asymmetric division process, triggers a profound remodeling of the overlying cortical region (hereafter referred to as the polarized cortex), including disappearance of membrane microvilli, formation of a thick F-actin cap and polarized accumulation of myosin II, PAR3, PAR6 and activated Rac GTPase (Longo and Chen, 1985; Maro et al., 1986; Simerly et al., 1998; Vinot et al., 2004; Duncan et al., 2005; Deng et al., 2007; Halet and Carroll, 2007). The mechanism and role of this cortical remodeling are not completely understood. Early studies have revealed the unique ability of meiotic chromosomes to induce cortical remodeling in a microtubule-independent manner (Longo and Chen 1985; Maro et al. 1986), and a recent report has uncovered the instrumental role of the GTPase Ran in this process (Deng et al., 2007). Ran is activated (Ran–GTP) by the GTP/GDP exchange factor RCC1, which is bound to chromatin, resulting in the generation of a Ran–GTP gradient centered on the meiotic chromosomes (Dumont et al., 2007). This diffusible Ran–GTP signal is necessary for meiotic chromosomes to induce, at a distance, remodeling of the nearby cortex, such as formation of the F-actin cap (Deng et al., 2007). However, the molecular cascade initiated by Ran to polarize the oocyte is poorly understood.

In other systems, from budding yeast to mammalian epithelia, cell polarization is frequently associated with polarized activation of the Rho GTPase Cdc42 (Heasman and Ridley, 2008). Interestingly, N-WASP, a Cdc42 effector involved in actin filament nucleation and branching (Miki et al., 1996; Rohatgi et al., 1999), was recently reported to accumulate in the polarized cortex of mouse MII oocytes (Yi et al., 2011). In addition, the Arp2/3 complex, a major actin filament nucleator and downstream effector of N-WASP, was shown to localize at the polarized cortex and to be required for the formation of the F-actin cap overlying the meiotic spindle (Yi et al., 2011; Sun et al., 2011). Overall, these findings point to a possible role for Cdc42 as the upstream activator of the N-WASP/Arp2/3 machinery in oocyte polarization. However, evidence for a polarized activation of Cdc42 in mammalian oocytes is currently missing.

To examine a possible role for Cdc42 activation in oocyte polarization, we employed a genetically-encoded fluorescent probe based on the Cdc42-binding domain of WASP (EGFP–wGBD; Benink and Bement, 2005) to detect active, GTP-bound Cdc42 in live mouse oocytes. We show that Cdc42–GTP accumulates, in a polarized fashion, in the cortex overlying the meiotic spindle, during both meiosis I and II. Furthermore, we found that the polarized activation of Cdc42 is driven by the Ran–GTP gradient generated by meiotic chromosomes, from metaphase through anaphase. In addition, we demonstrate that Cdc42 inhibition results in N-WASP detaching from the cortex, and the loss of the F-actin cap. We further show that this Cdc42/N-WASP/F-actin cascade is required for membrane protrusion and cytokinesis during polar body formation in meiosis II, but seems dispensable for emission of the first polar body. A defect in central spindle formation, associated with chromatid scattering, was also observed in activated MII oocytes lacking Cdc42 activation. Therefore, Cdc42 is a new protagonist in the molecular cascade leading to mammalian oocyte polarization and asymmetric division, lying downstream of the cytoplasmic Ran–GTP gradient, and upstream of N-WASP activation at the cortex.

## Materials and methods

### Oocyte collection and culture

MF1 mice (4–6 week-old) or OF-1 mice (8–10 week-old) were injected with 7–10 IU PMSG (Intervet, Milton Keynes, England, or Sigma, Lyon, France) for priming, followed 48 h later by 5–7.5 IU hCG (Intervet, Milton Keynes, England or Sigma, Lyon, France) to induce ovulation. GV and MII oocytes were collected from antral follicles and oviducts, respectively, and recovered in M2 medium. Oocytes were maintained at the GV stage by supplementing M2 medium with 250 μM dibutyryl-cAMP. For in vitro maturation, GV oocytes were cultured in M16 medium in a 5% CO2 incubator. To trigger oocyte activation and emission of the second polar body in a synchronized manner, MII oocytes were incubated for 8 min in M2 medium containing 7% ethanol, followed by wash, as described previously (Rogers et al., 2006). Ethanol treatment results in a single prolonged rise in cytosolic Ca^2+^ concentration, which recapitulates the events provoked by fertilization (Rogers et al., 2006). All reagents and media were from Sigma (Dorset, England, or Lyon, France), unless otherwise stated.

### Expression of EGFP–wGBD, Cdc42T17N, Cdc42Q61L, Rac1Q61L and RanT24N

Plasmids encoding the Cdc42–GTP probe EGFP–wGBD (a generous gift from Bill Bement) and dominant-negative Cdc42T17N (obtained from Gary Bokoch via Addgene, plasmid 12973) were subcloned in pcDNA3.1. EGFP–Cdc42Q61L in pcDNA3 and EGFP–Rac1Q61L in pcDNA3 were obtained from Gary Bokoch (Addgene, plasmids 12981 and 12986). After plasmid linearization, cRNAs were prepared in vitro using the mMessage mMachine T7 Ultra kit (Ambion) and pressure-injected in GV or MII oocytes. Expression of the EGFP–wGBD probe was not associated with significant dominant-negative phenotype in previous studies (Kim et al., 2000; Cannon et al., 2001; Benink and Bement, 2005; Ma et al., 2006; Zhang et al., 2008), nor in the present study. To inhibit Ran function, oocytes were injected with purified RanT24N protein (Cytoskeleton, Denver, CO, USA; 0.5 μg/μl in the pipette). Control oocytes were injected with an equivalent amount of water.

### Immunostaining and F-actin staining

Oocytes were fixed in paraformaldehyde (3.7% in PBS) for 30 min and permeabilized with Triton X100 (0.25% in PBS) for 20 min. After a 3-hour incubation in a block solution consisting of 3% BSA in PBS, oocytes were incubated with primary antibodies at 4 °C overnight, washed three times, then incubated with the secondary antibodies for 1 h at 37 °C. The following primary and secondary antibodies were used: N-WASP 30D10 (1:100; rabbit monoclonal, Cell Signaling Technology, Boston, MA, USA), alpha-tubulin (1:200; mouse monoclonal, Abcam, Paris, France), Alexa Fluor 488 Goat anti-mouse IgG, Alexa Fluor 488 Goat anti-rabbit IgG, and Alexa Fluor 555 Goat anti-rabbit IgG (all 1:1000; Life Technologies, Saint Aubin, France). To label F-actin, permeabilized oocytes were incubated for 5 min in PBS containing Alexa Fluor 546 Phalloidin (5 U/ml; Life Technologies), followed by wash. Chromatin was labeled with To-Pro-3 (5 μM; Life Technologies) in fixed oocytes, and with Hoechst 33342 (5 μg/ml; Life Technologies) in live oocytes.

### Image acquisition and analysis

Oocytes were placed on glass-bottom dishes (MatTek, Ashland, MA, USA) and imaged using either an LSM510meta confocal microscope (Carl Zeiss Ltd.) equipped with 365, 488 and 543-nm laser lines, or a Leica SP5 confocal microscope equipped with 488, 561 and 633-nm laser lines. For live oocyte imaging, temperature was maintained at 37 °C using an incubator fitted on the LSM510meta confocal microscope, or using a stage top incubator (model INUBG2E-GSI, Tokai Hit, Shizuoka-ken, Japan) fitted on the Leica SP5 confocal microscope. Images were processed using MetaMorph (Molecular Devices, Wokingham, UK). Fluorescence profiles were generated with the linescan function in MetaMorph. Fluorescence intensities were expressed as mean±S.E.M. and statistical analysis (*t*-test) was performed using GraphPad QuickCalcs (graphpad.com).

## Results

### Cdc42 is activated in cortical regions overlying meiotic chromosomes

**Fig. 1:**
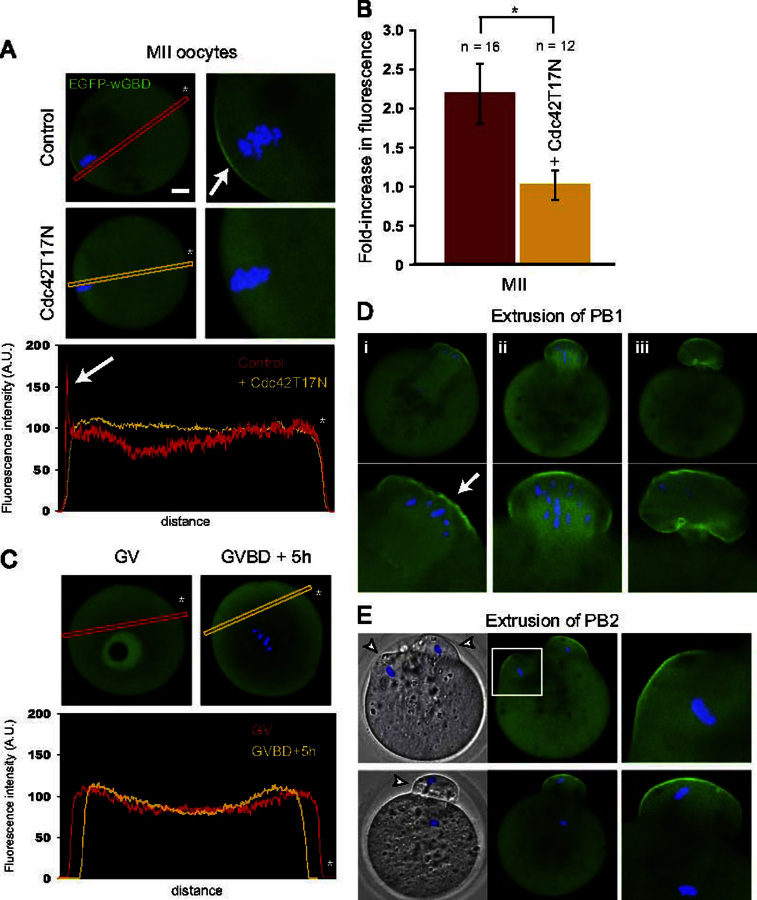
Polarized activation of Cdc42 in the cortex of mouse oocytes. Cdc42–GTP was detected using the EGFP–wGBD probe in live mouse oocytes at various stages of meiosis. (A) Top panels: Cdc42–GTP accumulates in the cortical region overlying the MII chromosomes. The right image is a magnification of the MII spindle area, with an arrow highlighting the polarized Cdc42–GTP cap. The fluorescence linescan profile corresponding to the red box is shown on the lower panel (red, Control), with an arrow pointing at the fluorescence spike corresponding to the Cdc42–GTP cap. The middle panels show an MII oocyte co-expressing EGFP–wGBD and Cdc42T17N. Note the absence of a Cdc42–GTP cap, resulting in a flat fluorescence profile (yellow, Cdc42T17N). Orientation of the linescans is indicated by the white asterisks. (B) Bar chart displaying the fold-increase in EGFP–wGBD fluorescence in the polarized cortex (overlying the MII chromosomes), relative to the opposite cortex, as measured using linescan analysis. Red: control MII oocytes. Yellow: MII oocytes expressing Cdc42T17N. (C) EGFP–wGBD distribution in GV-arrested (top left image) and prometaphase I (GVBD+5 h, top right) oocytes. Note the lack of detectable Cdc42 activation at the cortex, resulting in flat fluorescence profiles (lower panel). (D) Cdc42–GTP dynamics during PB1 emission. Note the accumulation of Cdc42–GTP in the cortex overlying one set of segregated chromosomes at an early stage of PB1 protrusion (i, arrow), and in the cortex of the protruding (ii) or fully emitted PB1 (iii). Bottom images are magnifications of the polar body area. (E) Cdc42–GTP dynamics during PB2 emission. The top panel shows an activated MII oocyte at an early stage of anaphase II. Note the two cortical protrusions (arrowheads on the brightfield image) overlying the two sets of chromatids, and enriched in Cdc42–GTP. The right image is a magnification of the protrusion delineated by the white square. The bottom panels show an activated MII oocyte at a late stage of PB2 protrusion. Note the accumulation of Cdc42–GTP in the cortex of the emitted PB2 (arrowhead in the brightfield image; magnification on the right). ^⁎^: *P*<0.001. Green: EGFP–wGBD. Blue: chromatin. Bar: 10 μm.

To examine Cdc42 activation, mouse oocytes were injected with cRNA encoding the Cdc42–GTP probe EGFP–wGBD (Benink and Bement, 2005). In previous studies, this probe was successfully used to detect localized Cdc42 activation in cancer cells (Kim et al., 2000), T cells (Cannon et al., 2001), and in the cortex of Xenopus oocytes during wound healing or polar body emission (Benink and Bement, 2005; Ma et al., 2006; Zhang et al., 2008). In mouse oocytes arrested at the MII stage, the probe accumulated in a restricted cortical area overlying the MII spindle (Fig. 1A, top panels, arrow). This polarized distribution generated a sharp spike on a linescan fluorescence profile (Fig. 1A, bottom panel, arrow), reaching a fluorescence intensity more than two-fold higher than in the opposite cortex (average fold-increase in fluorescence intensity against the opposite cortex: 2.18±0.38, *n*=16; Fig. 1B).

Inhibition of Cdc42 activation via the co-injection of cRNA encoding dominant-negative Cdc42T17N (Symons et al., 1996; Na and Zernicka-Goetz, 2006) resulted in partial or complete spindle detachment from the cortex, associated with a substantial reduction of the Cdc42–GTP cap, in a significant number of MII oocytes (*n*=7/18; data not shown). A similar spindle detachment was recently reported in oocytes treated with the Arp2/3 complex inhibitor CK-666, or following N-WASP knockdown (Yi et al., 2011). Spindle detachment effectively distances the chromatin from the cortex with the result that the chromatin-induced polarization is lost or reduced. To continue investigating the mechanism of Cdc42 action on cortical polarity, we therefore focussed on oocytes where the MII spindle had remained cortically-anchored, as exemplified in Fig. 1A (see also Fig. 3A). In these oocytes, Cdc42 inhibition (3–4 h following injection of cRNA encoding Cdc42T17N) resulted in the suppression of the cortical Cdc42–GTP cap (Fig. 1A, middle panels), and a flat fluorescence profile (fold-increase in fluorescence intensity: 1.01±0.19, *n*=12; Fig. 1A, bottom, and B).

To investigate the dynamics of Cdc42 activation during meiosis, we examined the distribution of the Cdc42–GTP probe in GV-arrested oocytes, and in oocytes undergoing maturation in vitro, following spontaneous meiosis resumption. In GV-stage oocytes, and in prometaphase I oocytes with a centrally-located spindle (GVBD+5 h), the probe remained cytosolic and no cortical accumulation could be detected (Fig. 1C). The probe was also enriched in the GV; however, this accumulation was insensitive to Cdc42 inhibition (data not shown), arguing for a non-specific phenomenon. After the spindle had reached the cortex and the metaphase I–anaphase I transition occurred, a distinct Cdc42–GTP cap could be detected in the cortical region overlying the segregated homologs (Fig. 1Di, arrow). During polar body protrusion (Fig. 1Dii) and until after cytokinesis (Fig. 1Diii), Cdc42–GTP remained enriched in the cortex of the first polar body. In the same way, in MII oocytes activated with ethanol to trigger anaphase II parthenogenetically, Cdc42–GTP accumulated in the two cortical protrusions overlying the two sets of segregated chromatids (Fig. 1E, top panels). At later stages, a single cortical protrusion remained and formed the second polar body, the cortex of which was enriched in Cdc42–GTP (Fig. 1E, bottom panels).

**Figure S1:**
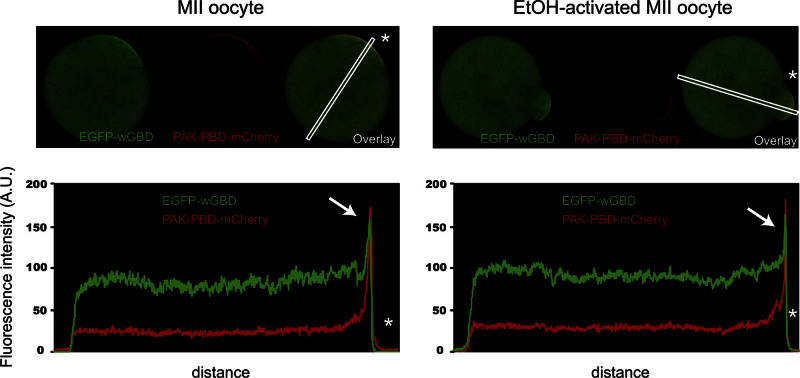
**EGFP–wGBD and PAK–PBD–mCherry co-localize in the polarized cortex.** MII oocytes were injected with cRNA encoding EGFP–wGBD and PAK–PBD–mCherry. Confocal images were acquired in MII oocytes (left panel; *n*=9) and in EtOH-activated MII oocytes at the time of PB2 protrusion (right panel; *n*=8). The yellow color indicates co-localization. Fluorescence linescan profiles corresponding to the white boxes are shown. The arrows indicate the co-localization of fluorescence peaks. Asterisks give the orientation of the profiles.

Altogether, these data suggest that in mouse oocytes undergoing the first or second meiotic division, Cdc42 is activated selectively in cortical regions overlying meiotic chromosomes or chromatids, with exactly the same dynamics as formation of the F-actin cap. This distribution is reminiscent of the polarized localization of the Rac–GTP probe PAK–PBD–YFP in regions overlying chromatin in mouse oocytes (Halet and Carroll, 2007). Oocytes expressing both EGFP–wGBD and PAK–PBD–mCherry exhibited strong co-localization of the two probes in the cortex overlying the MII spindle, and in the cortex overlying the chromatid cluster in PB2 (Fig. S1). These data suggest that oocyte polarization is associated with the activation of both Rac and Cdc42 in the cortex overlying meiotic chromatin.

### Polarized Cdc42 activation is driven by the Ran–GTP gradient

**Fig. 2:**
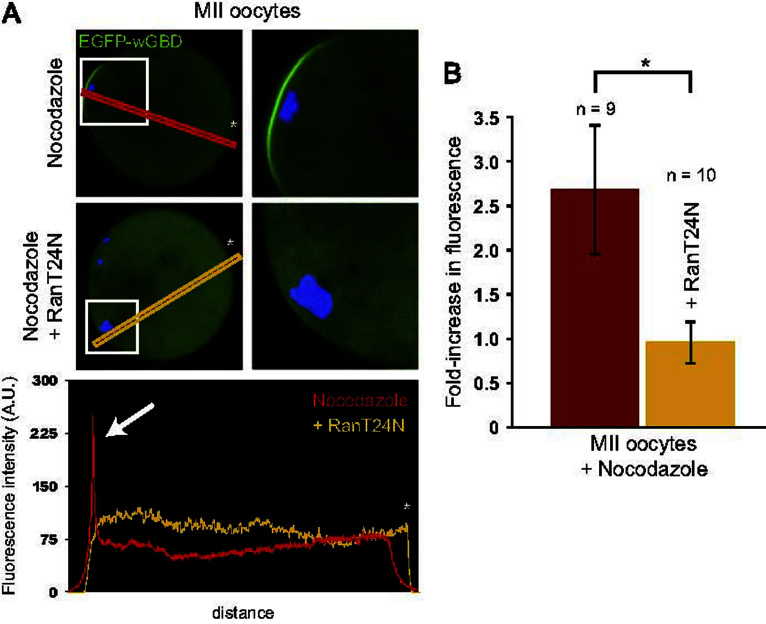
Polarized Cdc42 activation is Ran–GTP-dependent. MII oocytes expressing EGFP–wGBD were treated with nocodazole (1 μM for 30 min) in order to promote the formation of chromosome clusters apposed to the cortex. (A) Top panels: cortical Cdc42 activation (as detected by the localized accumulation of EGFP–wGBD) in the vicinity of a chromosome cluster. A magnified image is shown on the right. The corresponding increase in fluorescence is shown on the linescan analysis (bottom panel, white arrow). The middle panels show an MII oocyte injected with purified RanT24N. Note the absence of Cdc42 activation in the cortex overlying the chromatin mass, resulting in a flat fluorescence profile (bottom panel; RanT24N). Orientation of the linescans is indicated by the white asterisks. (B) Bar chart displaying the fold-increase in EGFP–wGBD fluorescence in the cortex overlying the chromosome cluster, relative to the opposite cortex devoid of chromosome cluster. Red: nocodazole-treated MII oocytes, injected with water. Yellow: nocodazole-treated MII oocytes injected with RanT24N. ^⁎^: *P*<0.001.

The above data indicate that Cdc42 activation occurs when meiotic chromatin is localized in the vicinity of the cortex. This is reminiscent of the chromatin-induced F-actin cap formation, which requires the chromatin-centered Ran–GTP gradient (Longo and Chen, 1985; Maro et al., 1986; Deng et al., 2007). To address a direct role for meiotic chromatin in inducing Cdc42 activation, we treated MII oocytes with the microtubule poison nocodazole, a procedure which results in the formation of chromosome clusters closely associated with the cortex (Maro et al., 1986). As seen in Fig. 2A, a Cdc42–GTP cap was maintained in the cortex overlying the MII chromosome clusters (fold-increase in fluorescence intensity against the opposite cortex devoid of clusters: 2.67±0.72, *n*=9; Fig. 2B), arguing for a direct relationship between chromosome positioning and Cdc42 activation in the nearby cortex. This chromatin-induced Cdc42 activation was prevented by co-expression of Cdc42T17N (data not shown). To investigate the contribution of the Ran GTPase, we inhibited Ran–GTP production by injecting oocytes with purified dominant-negative RanT24N (Dasso et al., 1994). Because interfering with Ran function disturbs MII spindle integrity (Dumont et al., 2007a), and therefore could affect chromosome positioning, we used nocodazole-treated oocytes to ensure that chromosome clusters remained apposed to the cortex. In these conditions, the Cdc42–GTP cap was abolished, despite the close proximity between chromatin clusters and the oocyte cortex (fold-increase in fluorescence intensity against the opposite cortex: 0.95±0.24, *n*=10; Fig. 2A and B). These data suggest that Cdc42 activation is controlled by the Ran–GTP gradient emanating from chromatin, providing an explanation for the restricted distribution of Cdc42–GTP in cortical regions overlying meiotic chromosomes.

### Cdc42–GTP recruits N-WASP to the polarized cortex

**Fig. 3:**
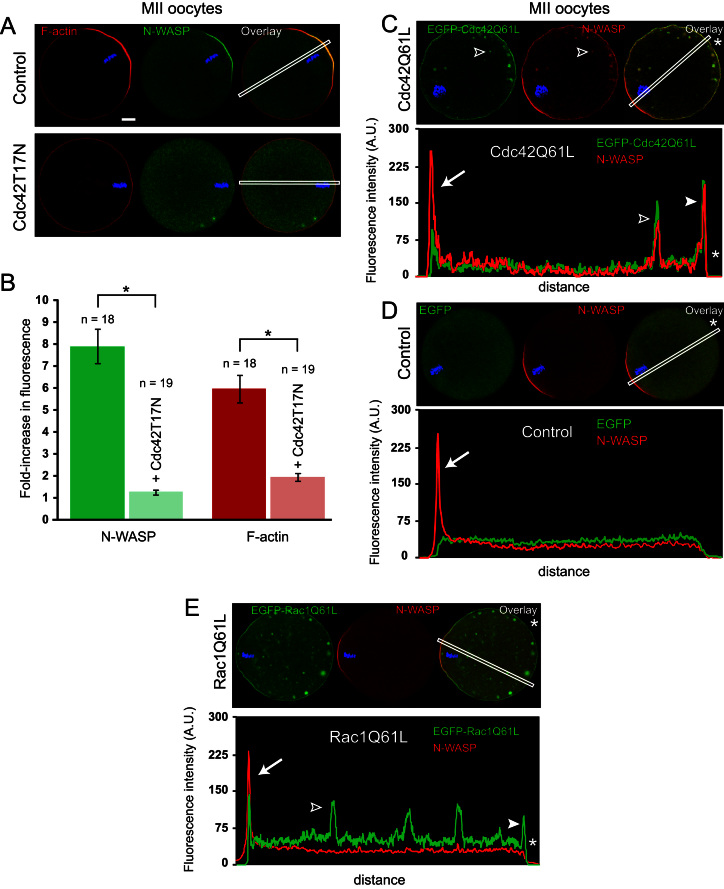
Cdc42–GTP controls N-WASP localization at the cortex. MII oocytes were fixed and examined for endogenous N-WASP (immuno-staining) and actin filament (Alexa Fluor 546-phalloidin staining) distribution. (A) In control, water-injected MII oocytes, N-WASP co-localizes with the polarized F-actin cap (top panel; co-localization appears in yellow). In MII oocytes expressing Cdc42T17N, N-WASP is cytosolic and the F-actin cap is strongly decreased (lower panel). (B) Bar chart displaying the fold-increase in N-WASP (green bars) or F-actin (red bars) fluorescence in the cortex overlying the MII chromosomes, relative to the opposite cortex. The data are based on fluorescence profiles generated across the oocyte diameter, as shown by the white boxes in (A). Control oocytes are in dark green/red; oocytes expressing Cdc42T17N are in light green/pink. *: *P*<0.001. (C) In oocytes expressing constitutively-active EGFP–Cdc42Q61L, N-WASP staining re-distributes around the oocyte cortex and on cytoplasmic vesicles, co-localized with EGFP–Cdc42Q61L (co-localization is in yellow on the overlay image). The open arrowhead points to a cytoplasmic vesicle positive for N-WASP and EGFP–Cdc42Q61L staining. The corresponding fluorescence profile is shown, where the arrow points at the cortex overlying the MII chromosomes, the closed arrowhead points at the opposite cortex, and the open arrowhead points at the cytoplasmic vesicle mentioned above. Note the overlapping fluorescence spikes showing co-localization of N-WASP with EGFP–Cdc42Q61L. (D) N-WASP staining is restricted to the polarized cortex overlying the MII chromosomes in control oocytes expressing EGFP alone. The fluorescence profile corresponding to the white box is shown, with an arrow pointing at the cortical N-WASP accumulation over the MII chromosomes. (E) In oocytes expressing constitutively-active EGFP–Rac1Q61L, N-WASP remains enriched in the polarized cortex overlying the MII chromosomes, and does not redistribute around the cortex, nor on cytoplasmic vesicles, where EGFP–Rac1Q61L is enriched. On the corresponding fluorescence profile, the arrow points at the cortex overlying the MII chromosomes, the closed arrowhead points at the opposite cortex, and the open arrowhead points at a cytoplasmic vesicle. Note the absence of N-WASP on these last two structures. Orientation of the linescans is indicated by the white asterisks. Bar: 10 μm.

The polarized accumulation of Cdc42–GTP in the oocyte cortex and in protruding polar bodies, suggests a contribution of the GTPase to cortical polarization, which is exemplified by the formation of an F-actin cap. In this regard, N-WASP is a strong candidate as a Cdc42 effector, since it links Cdc42 activation to actin filament nucleation and branching, via the activation of the Arp2/3 complex (Rohatgi et al., 1999). Consistent with a recent study (Yi et al., 2011), immuno-labeling experiments revealed a prominent accumulation of endogenous N-WASP in the cortex overlying the MII spindle, co-localized with the F-actin cap (Fig. 3A, upper panel). Remarkably, inhibition of Cdc42 activation with Cdc42T17N abolished the cortical localization of N-WASP (Fig. 3A, lower panel, and B). Interestingly, the loss of cortical N-WASP was accompanied by a selective loss of the F-actin cap, while the basal cortical F-actin layer remained unaffected (Fig. 3A, lower panel, and B).

To confirm a key role for Cdc42–GTP in controlling N-WASP localization, we examined whether constitutively-active Cdc42 could affect N-WASP distribution. We expressed constitutively-active Cdc42Q61L tagged with EGFP (EGFP–Cdc42Q61L) in MII oocytes. As shown in Fig. 3C (representative of 12 similar observations), the constitutively-active GTPase was broadly distributed around the oocyte cortex, and was also detectable on cytoplasmic vesicles, presumably of endocytic origin (open arrowhead). Endogenous N-WASP co-localized with EGFP–Cdc42Q61L both in the cortex and on the cytoplasmic vesicles (Fig. 3C). This colocalization is highlighted in the linescan fluorescence profile displayed in Fig. 3C, which passes through a cytoplasmic vesicle (open arrowhead). In contrast, in control oocytes injected with cRNA encoding EGFP alone, N-WASP localized exclusively to the polarized cortex and was not detected in the rest of the cortex, nor on cytoplasmic structures (Fig. 3D; representative of 15 similar observations). We also tested if active Rac was a binding partner for endogenous N-WASP. As shown in Fig. 3E (representative of 10 similar observations), N-WASP localized exclusively to the polarized cortex in MII oocytes expressing constitutively-active EGFP–Rac1Q61L. Though constitutively-active Rac1 distributed all over the cortex and on cytoplasmic vesicles, as seen with Cdc42Q61L, these structures were not associated with an enrichment in N-WASP in the case of RacQ61L (see the fluorescence profile in Fig. 3E, where the closed arrowhead points at the opposite cortex, and the open arrowhead points at a cytoplasmic vesicle). Together, these data suggest that in mouse oocytes, active Cdc42 is the preferred binding partner for driving N-WASP localization.

Since Cdc42 acts in synergy with the phosphoinositide PIP2 to localize and activate N-WASP (Prehoda et al., 2000; Rohatgi et al., 2000; Padrick and Rosen, 2010), the accumulation of N-WASP in the polarized cortex could be a consequence of a local enrichment in this phosphoinositide species. However, we have shown in a previous study that PIP2 is not enriched in the polarized cortex of MII oocytes. Rather, the amicrovillar polarized cortex shows a relatively low PIP2 abundance compared with the rest of the cortex, rich in microvilli (Halet et al., 2002). Therefore, we propose that polarized Cdc42–GTP is the major determinant of N-WASP distribution and activation in the mammalian oocyte cortex.

### A polarized Cdc42/N-WASP/F-actin cascade is activated in meiosis I, but is dispensable for PB1 emission

**Fig. 4:**
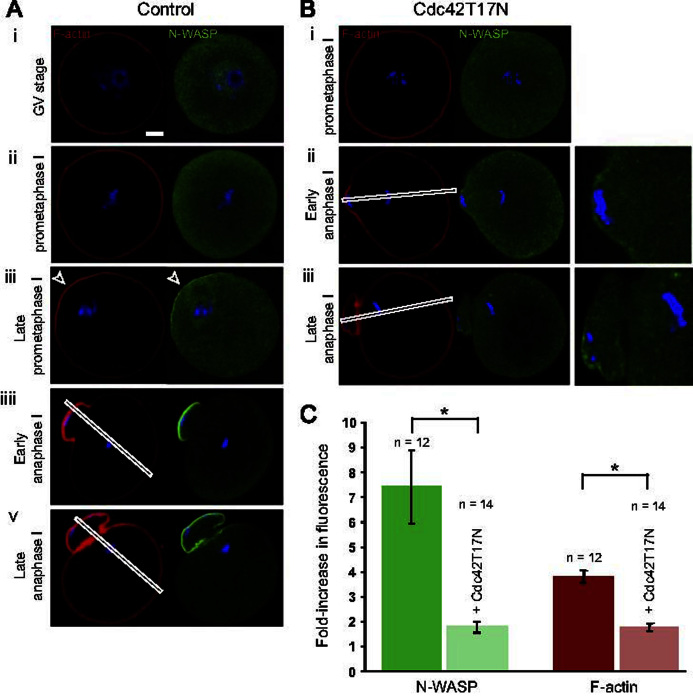
Cdc42 controls N-WASP and F-actin accumulation in the polar body in meiosis I. (A) N-WASP (immuno-staining) and F-actin (Alexa Fluor 546-phalloidin staining) distribution was examined at successive stages of meiosis resumption: GV arrest (i), prometaphase I (GVBD+5 h, ii), late prometaphase I (GVBD+7/8 h, iii), early anaphase I (iiii), late anaphase I (v). Note the enrichment of N-WASP and F-actin in the cortex overlying meiotic chromosomes (arrowhead in iii) and in the cortex of the protruding first polar body (iiii, v). (B) Oocytes expressing Cdc42T17N were examined at 7.5/8 h post-GVBD. Thirty-six per cent of these oocytes exhibited a centrally-located spindle apparatus (i) and lacked any cortical N-WASP. The remaining oocytes had reached anaphase I, and were observed at early (ii) or late stages (iii) of polar body protrusion. Note the decrease in N-WASP and F-actin staining in the protruding polar bodies (see Aiiii and Av for a comparison). Magnified images of N-WASP immuno-labeling in the polar body region are shown on the right. (C) Bar chart displaying the fold-increase in N-WASP (green bars) or F-actin (red bars) fluorescence in the cortex of the protruding PB1, relative to the opposite cortex. The data are based on fluorescence profiles generated across the oocyte diameter, as shown by the white boxes in Aiiii, v and Bii, iii. Data from oocytes at early anaphase I and late anaphase I were pooled to generate the bar graph. Control oocytes are in dark green/red; oocytes expressing Cdc42T17N are in light green/pink. ^⁎^: *P*<0.001. Confocal images are representative of 9–16 similar observations. Bar: 10 μm.

In order to investigate whether the proposed Cdc42/N-WASP pathway contributes to the establishment of oocyte polarization, we examined N-WASP distribution at successive stages of oocyte maturation. In GV-stage oocytes, and in prometaphase I oocytes with a centrally-located spindle, N-WASP was essentially cytosolic and no cortical accumulation was detectable (Fig. 4Ai and ii). This is consistent with the finding that these oocytes also lack any detectable Cdc42 activation at the cortex (Fig. 1C). Also in agreement with these findings is the observation that cortical F-actin staining was uniformly distributed in the oocyte cortex (Fig. 4Ai and ii). In contrast, as the MI spindle reached the cortex (GVBD+7/8 h, late prometaphase I), N-WASP started to accumulate in the cortical region overlying the spindle, coincident with the formation of a polarized actin cap (Fig. 4Aiii, arrowhead). During PB1 protrusion (Fig. 4Aiiii, early anaphase I), until after complete PB1 emission (Fig. 4Av, late anaphase I), N-WASP remained exclusively enriched in the cortex of the polar body, co-localized with a dense F-actin staining. These data suggest that, similar to our observations in MII-arrested oocytes (Fig. 3), meiotic chromosomes are capable of inducing, at a distance, N-WASP accumulation in the overlying cortex. This is consistent with our finding that Cdc42–GTP accumulates selectively in the cortex overlying the chromosomes during meiosis I (Fig. 1D).

In the light of these findings, we examined the effects of Cdc42 inhibition on the rate of PB1 emission in oocytes undergoing in vitro maturation. Consistent with previous studies (Na and Zernicka-Goetz, 2006; Cui et al., 2007; Bielak-Zmijewska et al., 2008), we found that 48% (*n*=49/103 oocytes) of oocytes expressing Cdc42T17N failed to emit PB1 following a 16-hour culture period. Staining for chromosomes and tubulin revealed that the spindle was centrally-located (data not shown), consistent with the defect in spindle migration previously reported (Na and Zernicka-Goetz, 2006; Cui et al., 2007). Another 18% (*n*=19/103) cleaved symmetrically to produce a polar body indistinguishable from the oocyte. The remaining 34% (35/103 oocytes) matured to MII and extruded a small polar body. During the same culture period, 92% (44/48 oocytes) of control, water-injected oocytes, extruded a small PB1 and arrested at MII (data not shown).

The observation that a third of oocytes expressing Cdc42T17N succeeded to emit a small PB1 was puzzling, as it suggested that Cdc42 was not inhibited. To investigate this issue, oocytes expressing Cdc42T17N were fixed at the time of polar body emission (GVBD+7.5/8 h, as determined using time-matched control oocytes) and processed for N-WASP immuno-staining. As mentioned above, a significant proportion of these oocytes (*n*=9/25, 36%) exhibited a centrally-located MI spindle (as evidenced by chromosome labeling), and lacked any cortical N-WASP (Fig. 4Bi). The remaining oocytes (*n*=16/25) had proceeded to anaphase I and were observed at early (Fig. 4Bii) or late (Fig. 4Biii) stages of PB1 emission. Interestingly, N-WASP was virtually depleted from the cortex of these protruding polar bodies, despite close apposition of chromosomes to the cortex (Fig. 4Bii,iii). The enrichment in polymerized F-actin was also substantially decreased (Fig. 4Bii and iii). To quantify these data, we performed linescan fluorescence intensity measurements (like those shown in Fig. 1) along the axis of the anaphase I spindle, and we measured the increase in N-WASP or F-actin staining in the cortex of the polar body, relative to the opposite oocyte cortex. These measurements, summarized in the bar chart in Fig. 4C, revealed a four-fold decrease in N-WASP accumulation, and a two-fold decrease in F-actin staining in the cortex of the protruding polar bodies, following Cdc42 inhibition.

**Figure S2:**
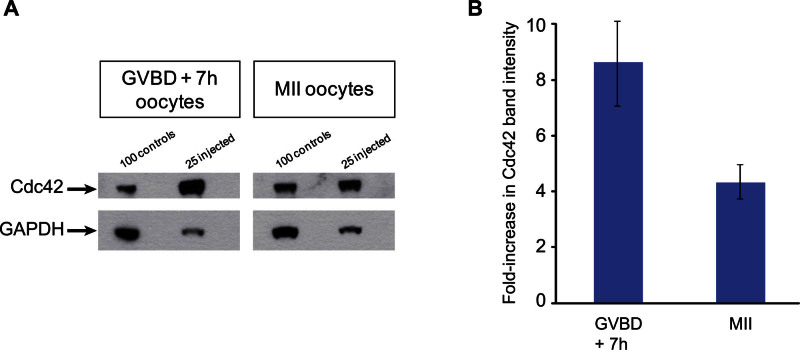
**Quantification of Cdc42T17N expression by Western blot. (A)** Metaphase II oocytes (MII) and GV oocytes were injected with cRNA encoding Cdc42T17N. Lysates were prepared from 25 MII oocytes after three hours of expression (25 injected, right panel), and from 25 oocytes at GVBD+7 h (25 injected, left panel), to match the functional experiments. For controls, lysates were prepared from 100 oocytes in order to increase the reliability of the signal (100 controls). GAPDH detection was used as a loading control, and to normalize the Cdc42 band intensities. A typical experiment is shown (*n*=3). (B) Bar graph showing the relative increase in Cdc42 band intensity in oocytes expressing Cdc42T17N. The results from three experiments were averaged. GVBD+7 h oocytes : 8.64±1.56. MII oocytes : 4.33±0.62.

To ensure that Cdc42T17N was effectively expressed in mouse oocytes undergoing PB1 emission, the amount of Cdc42 protein was quantified in oocyte lysates using the Western blot technique. These experiments revealed that injected oocytes expressed a ∼7-fold excess of Cdc42T17N over endogenous Cdc42, at the time of PB1 formation (GVBD+7 h; Fig. S2). In comparison, MII oocytes expressing the Cdc42T17N cRNA for three hours contained a ∼three-fold excess of Cdc42T17N over endogenous Cdc42 (Fig. S2), which is sufficient to prevent N-WASP activation and formation of the F-actin cap (Fig. 3A). Therefore, it can be argued that oocytes examined at GVBD+7 h express sufficient levels of Cdc42T17N to fully inhibit Cdc42 signaling.

Altogether, these results suggest that Cdc42 inhibition is effective, and precludes N-WASP recruitment at the cortex, in oocytes undergoing PB1 protrusion. These data therefore argue for the existence of a compensatory mechanism driving polar body protrusion in the absence of a functional Cdc42/N-WASP pathway. Alternatively, a minute amount of cortical N-WASP and a highly diminished F-actin cap may suffice to support polar body protrusion in meiosis I.

### Cdc42 inhibition in activated oocytes prevents emission of PB2 and induces central spindle defects

**Fig. 5:**
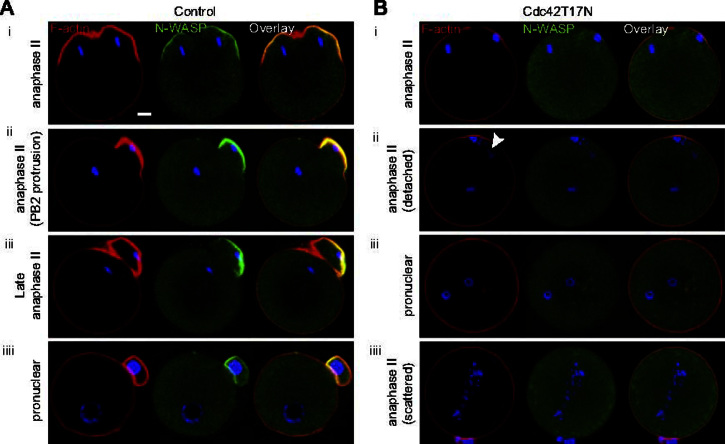
Cdc42 activation is required for PB2 emission in activated oocytes. (A) Ethanol-activated MII oocytes were examined for F-actin and N-WASP staining at successive stages of PB2 emission: early anaphase II (i), PB2 protrusion (ii), late anaphase II (fully emitted PB2, iii), and pronuclear stage (iiii). Note the enrichment of N-WASP in F-actin-rich cortical protrusions overlying the chromatid masses in (i) and (ii). (B) MII oocytes expressing Cdc42T17N were activated with ethanol and examined for F-actin and N-WASP staining at early anaphase II (i and ii) and at the time of pronucleus formation (iii, iiii). Note the absence of cortical N-WASP at all stages, and the lack of polar body protrusion. In (ii), note the perpendicular orientation of the spindle apparatus and the chromatid scattering (arrowhead). Bar: 10 μm.

We next investigated N-WASP dynamics and the relevance of the Cdc42/N-WASP/F-actin cascade during PB2 emission in activated MII oocytes. In control conditions, N-WASP accumulated symmetrically in cortical protrusions overlying the two sets of segregated chromatids, and later became restricted to the polar body-forming protrusion (Fig. 5Ai–iii; *n*=10–12 similar observations). Polarized F-actin co-localized with N-WASP and followed essentially the same dynamics (Fig. 5Ai–iii). At the pronuclear stage (∼5 h post-activation), F-actin was uniformly distributed in the oocyte cortex, and N-WASP was cytosolic in the oocyte (Fig. 5Aiiii; representative of 11 similar observations). These data are consistent with a chromatin-centered diffusible signal driving N-WASP accumulation in the cortex overlying the chromatids. In support of this model, and in agreement with a recent study (Yi et al., 2011), nocodazole-induced chromosome clusters induced N-WASP and F-actin caps in the nearby cortex, that were inhibited by dominant-negative RanT24N (data not shown). Overall, these data suggest that N-WASP and F-actin dynamics in anaphase II are controlled by the Ran–GTP gradients emanating from each set of segregated chromatids.

In oocytes expressing Cdc42T17N, the great majority (47/55 oocytes; 85%) failed to extrude a polar body in the two hours following ethanol activation (against 94% of PB2 emission in control oocytes). Examination of N-WASP dynamics revealed that the Cdc42 effector remained cytosolic throughout the activation process, and F-actin-rich protrusions were absent, despite close apposition of chromatids to the cortex (Fig. 5Bi, *n*=14; see Fig. 5Ai for a comparison with controls). Some oocytes (*n*=4) showed anaphase figures oriented perpendicular to the cortex, which could be due to the MII spindle detaching from the cortex before the oocytes were challenged with ethanol. In these oocytes, the chromatid mass apposed to the oocyte cortex also failed to induce the recruitment of N-WASP and the formation of an F-actin-rich protrusion (Fig. 5Bii, *n*=4; see Fig. 5Aii for a comparison). These observations suggest that Cdc42 signaling was effectively inhibited by Cdc42T17N, precluding N-WASP binding to the cortex and the formation of F-actin-rich protrusions in anaphase II, ultimately resulting in a failure to emit PB2.

**Fig. 6:**
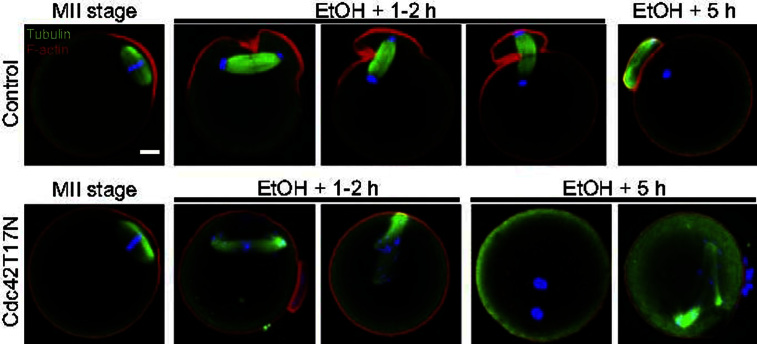
Cdc42 inhibition induces central spindle and cytokinesis defects in activated oocytes. MII oocytes were examined for F-actin and tubulin staining during the MII arrest (MII stage), 1–2 h after ethanol activation (EtOH+1–2 h), and at the time of pronucleus formation (EtOH+5 h). Upper panels: control oocytes, injected with water. Note the robust and strictly bipolar central spindle. Lower panels: oocytes expressing Cdc42T17N. Note the chromatid scattering and spindle distorsion at 1–2 h and 5 h post-activation. At 5 h post-activation, the majority of oocytes expressing Cdc42T17N were binucleated (left image) or blocked in meiosis with a distorted spindle (right image). Images of EtOH-activated oocytes are compressed Z-stacks of 2–8 images, in order to capture the entire spindle structure. Red: F-actin. Green: tubulin. Blue: chromatin. Bar: 10 μm.

Activated MII oocytes expressing Cdc42T17N were next examined for pronucleus formation five hours after activation. A majority of these oocytes (13/22 oocytes; 59%) were binucleated, indicating that, consistent with a failure to form F-actin-rich protrusions necessary for PB2 formation, these oocytes failed to undergo cytokinesis (Fig. 5Biii). Another third of the oocytes (8/22; 36%) failed to exit meiosis and to form pronuclei, but instead exhibited chromatid scattering across the cytosol (Fig. 5Biiii). To examine the integrity of the spindle, oocytes injected with water (controls) or expressing Cdc42T17N were processed for tubulin immuno-staining. In controls, the robust central spindle formed in anaphase remained strictly bipolar for the duration of the PB2 emission process (Fig. 6A top row, EtOH+1–2 h), and 86% (*n*=45/52 oocytes) of oocytes examined at 5 h post-activation exhibited a second polar body and a single pronucleus (Fig. 6A top row, EtOH+5 h). In oocytes expressing Cdc42T17N, chromatid scattering and spindle distorsion were apparent in a fraction of oocytes (*n*=9/30) examined 1–2 h following ethanol activation (Fig. 6A bottom row; EtOH+1–2 h; see also Fig. 5Bii, arrowhead). At 5 h post-activation (Fig. 6A bottom row, EtOH+5 h), Cdc42T17N-expressing oocytes were mainly binucleated (*n*=15/28) or arrested in meiosis with a distorted spindle and scattered chromatids (*n*=9/28). Thus, in addition to causing defective cortical polarization and a failure to emit PB2, Cdc42 inhibition also interfered with the formation of a functional central spindle capable of supporting chromatid segregation and cytokinesis during meiosis resumption in activated oocytes.

## Discussion

The development of an F-actin cap in the cortex overlying the metaphase spindle is a conserved feature of oocyte polarization in mammals, amphibians and lower invertebrates (Longo and Chen, 1985; Maro et al., 1986; Sardet et al., 1992; Zhang et al., 2008). In mouse oocytes, the thick F-actin cap overlying the MII spindle was first described over 25 years ago (Longo and Chen, 1985; Maro et al., 1986), but it is only recently that mechanistic insights were provided, through the finding that the F-actin cap is induced by the Ran–GTP gradient generated by meiotic chromosomes (Deng et al., 2007). The same group has recently reported that the machinery for actin filament nucleation, comprising the Arp2/3 complex and its upstream regulator N-WASP, also localized in the polarized cortex in a Ran–GTP-dependent manner, and was responsible for generating a flow of actin filaments and cytoplasmic streaming necessary to maintain the MII spindle in its eccentric location (Yi et al., 2011). Therefore, the next challenge in our understanding of mammalian oocyte polarization and spindle positioning, is to identify the mechanism by which Ran–GTP promotes the polarized activation of the N-WASP/Arp2/3 machinery in the oocyte cortex (Verlhac, 2011).

We now introduce Cdc42 as a new protagonist in the molecular cascade leading to oocyte polarization. Through the direct monitoring of Cdc42–GTP, we describe for the first time the polarized activation of Cdc42 in the cortex overlying the meiotic spindle in mouse oocytes (Fig. 1). Our data suggest that the polarized activation of Cdc42 is driven by the Ran–GTP gradient emanating from meiotic chromosomes, and serves to recruit N-WASP at the cortex to build the F-actin cap. According to the current model, N-WASP activation most certainly involves coincident binding to Cdc42–GTP and PIP2 in the plasma membrane, to relieve autoinhibition (Padrick and Rosen, 2010). However, a direct contribution of PIP2 in the polarized recruitment of N-WASP is unlikely, since our previous work has shown that PIP2 is not enriched in the polarized cortex of MII oocytes (Halet et al., 2002). Thus, we propose the following signaling cascade for mouse oocyte polarization:

Chromosomes→Ran→Cdc42→N-WASP−Arp2/3→F-actincap

The same signaling cascade operates during anaphase, resulting in the formation of F-actin-rich protrusions overlying the segregated chromosomes (anaphase I) or chromatids (anaphase II). Experiments using Cdc42T17N to disrupt Cdc42 activation and N-WASP localization, suggest that this signaling cascade is essential for the emission of PB2 in activated MII oocytes. One attractive hypothesis is that N-WASP-driven actin filament nucleation and branching provides the protrusive force necessary for membrane deformation around the segregated chromatids, to form the polar body (Condeelis, 1993; Pollard and Borisy, 2003). Considering that cortical tension, which is dependent on F-actin, is increased almost 3-fold in the polarized amicrovillar cortex of MII oocytes (Jégou et al., 2008; Larson et al., 2010), it is tempting to assume that the polarized Cdc42–GTP/N-WASP pathway also serves to prevent the collapse of the second polar body, by maintaining a thick cortical F-actin layer and increased cortical rigidity in the protruded membrane. Further investigations will be necessary to elucidate how the actin filaments forming the polarized F-actin cap in MII oocytes can fulfill these multiple roles – i.e., actin flow, cortical tension and membrane protrusion – and how this is regulated in space and time during the meiotic cell cycle.

There is increasing evidence that Cdc42, beyond its ubiquitous function as a regulator of actin dynamics, could be involved in spindle and chromosome dynamics during the cell cycle. In mitosis, Cdc42 inhibition was shown to result in abnormal chromosome segregation, due to defective kinetochore–microtubule attachments and chromosome congression in metaphase (Yasuda et al., 2004; Oceguera-Yanez et al., 2005). Consistent with a role for Cdc42 in regulating spindle dynamics, Cui et al. (2007) reported that injection of siRNA against Cdc42 resulted in spindle defects in MII oocytes. Apart from occasional spindle detachment, we did not notice obvious defects in spindle shape or chromosome alignment in MII oocytes, following acute Cdc42 inhibition with Cdc42T17N (Figs. 1, 3 and 6). However, we cannot exclude the possibility that chromosome attachment to kinetochore microtubules was defective, but remained unnoticed. The integrity of the central spindle in activated oocytes was, however, strongly affected (Fig. 6). Though the molecular basis of this defect is unknown at this time, these data point at a possible role for Cdc42 in promoting central spindle assembly and/or stability in anaphase II. Interestingly, spindle distorsion during anaphase II, and a failure to exit meiosis, were also reported in oocytes with decreased cortical tension, consecutive to expression of dominant-negative radixin (Larson et al., 2010). Thus, central spindle distorsion in anaphase II could represent a stereotypical response to defective cortical remodeling and unbalanced cortical forces during PB2 emission.

Inhibition of Cdc42 signaling using dominant-negative or constitutively-active Cdc42 mutants, RNA interference or treatment with Toxin B, has previously been shown to decrease the rate of polar body emission during the first meiotic division (Na and Zernicka-Goetz, 2006; Cui et al., 2007; Bielak-Zmijewska et al., 2008). In all these studies however, inhibition was incomplete, as a substantial proportion (30–40%) of oocytes still managed to emit PB1, raising the idea of a compensatory mechanism. In the present study, we confirm the partial inhibitory effect of Cdc42T17N on PB1 emission, possibly due to spindle migration defects (Na and Zernicka-Goetz, 2006). We further show that the remaining oocytes, which succeeded in protruding PB1, could do so in the absence of N-WASP activation at the polarized cortex (Fig. 4). Therefore, the requirement for Cdc42/N-WASP activation is not as stringent during PB1 emission as it is during PB2 emission, and we suggest that an alternative mechanism supports membrane deformation and PB1 protrusion in the absence of Cdc42/N-WASP signaling. It is noteworthy that, contrary to the F-actin cap, the basal cortical F-actin layer is independent of Cdc42 (this study), N-WASP (Yi et al., 2011) or Arp2/3 activation (Yi et al., 2011; Sun et al., 2011). Therefore, alternative protrusive forces may be provided by actin filaments generated by a machinery other than N-WASP/Arp2/3. The Fmn2-nucleated cytoplasmic actin cables, which drive meiotic spindle migration towards the cortex, have been suggested to generate pushing forces, and therefore could possibly contribute to membrane deformation in anaphase I (Li et al., 2008; Azoury et al., 2011). Fmn2-nucleated actin filaments may also occur at the cortex and participate in PB1 protrusion, since Fmn2 was shown to localize at the oocyte cortex (Azoury et al., 2011). Inflation of the polar body may also be facilitated by elevated hydrostatic pressure in the vicinity of the polar body-forming area, consecutive to elevated acto–myosin contractility during anaphase (Condeelis, 1993; Charras et al., 2005). In contrast, the contribution of other Rho GTPases is unlikely, since PB1 emission can still occur in oocytes treated with toxin B, a broad inhibitor of Rho GTPases (Bielak-Zmijewska et al., 2008). The existence of an alternative mechanism independent of Cdc42/N-WASP is also supported by the work of Dumont et al. (2007), who demonstrated that PB1 emission was unaffected by the inhibition of Ran during meiosis I. According to our model, polarized activation of the Cdc42/N-WASP/F-actin pathway should be absent, or strongly diminished, in these oocytes. Our data suggest however that the lack of Cdc42/N-WASP signaling in activated MII oocytes cannot be compensated for by alternative mechanisms.

In a search for the mechanism coupling spindle positioning to polar body formation in Xenopus eggs, Ma et al. (2006) described a localized activation of Cdc42 at the site where the spindle pole interacts with the cortex during meiosis I. This localized activation of Cdc42 was strictly dependent on the perpendicular orientation of the spindle relative to the cortex, and it was suggested that spindle pole-associated proteins may promote Cdc42 activation at the cortex (Zhang et al., 2008). Our data in mouse oocytes contrast with the frog egg model. Firstly, we observed a robust activation of the Cdc42/N-WASP/F-actin pathway in metaphase II-arrested oocytes, where the spindle lies parallel to the cortex. Secondly, we show that the spatial cue for polarized Cdc42 activation is provided by the chromosome-centered Ran–GTP gradient, in a microtubule-independent manner. Thus, amphibian and mammalian oocytes have developed distinct strategies for controlling the polarized activation of Cdc42 necessary for polar body protrusion. Consistent with a Ran-dependent, but microtubule-independent mechanism in mouse oocytes, sperm chromatin, which is not associated with microtubules, is also capable of inducing polarization of the nearby cortex in fertilized mouse oocytes, associated with the protrusion of the so-called fertilization cone (Simerly et al., 1998; Deng and Li, 2009). We speculate that protrusion of the fertilization cone also proceeds via the localized activation of the Cdc42/N-WASP/F-actin pathway, under the control of the Ran–GTP gradient generated by paternal chromatin.

Altogether, the results presented above support a key role for Cdc42 in the molecular cascade leading to mouse oocyte polarization and protrusion of the second polar body. We have shown that Cdc42 is activated in a polarized fashion in the oocyte cortex, under the control of the chromatin-driven Ran–GTP gradient. Cdc42 activation is essential for polarized N-WASP recruitment and the building of the F-actin caps overlying the clusters of meiotic chromosomes or chromatids. The mechanism by which Ran–GTP promotes Cdc42 activation is still elusive at this point, and deserves further investigations. It may involve the release of a cargo protein complexed with importins, and/or the polarized recruitment and activation of a yet-to-be identified Cdc42–GTP/GDP exchange factor.

## Footnotes

Appendix A Supplementary data associated with this article can be found in the online version at doi:10.1016/j.ydbio.2013.01.029.

## References

[bib1] Azoury J., Lee K.W., Georget V., Rassinier P., Leader B., Verlhac M.H. (2008). Spindle positioning in mouse oocytes relies on a dynamic meshwork of actin filaments. Curr. Biol..

[bib2] Azoury J., Lee K.W., Georget V., Hikal. P., Verlhac M.H. (2011). Symmetry breaking in mouse oocytes requires transient F-actin meshwork destabilization. Development.

[bib3] Benink H.A., Bement W.M. (2005). Concentric zones of active RhoA and Cdc42 around single cell wounds. J. Cell Biol..

[bib4] Bielak-Zmijewska A., Kolano A., Szczepanska K., Maleszewski M., Borsuk E. (2008). Cdc42 protein acts upstream of IQGAP1 and regulates cytokinesis in mouse oocytes and embryos. Dev. Biol..

[bib5] Cannon J.L., Labno C.M., Bosco G., Seth A., McGavin M.H., Siminovitch K.A., Rosen M.K., Burkhardt J.K. (2001). WASP recruitment to the T cell:APC contact site occurs independently of Cdc42 activation. Immunity.

[bib6] Charras G.T., Yarrow J.C., Horton M.A., Mahadevan L., Mitchison T.J. (2005). Non-equilibration of hydrostatic pressure in blebbing cells. Nature.

[bib7] Condeelis J. (1993). Life at the leading edge: the formation of cell protrusions. Annu. Rev. Cell Biol..

[bib8] Cui X.S., Li X.Y., Kim N.H. (2007). Cdc42 is implicated in polarity during meiotic resumption and blastocyst formation in the mouse. Mol. Reprod. Dev..

[bib9] Dasso M., Seki T., Azuma Y., Ohba T., Nishimoto T. (1994). A mutant form of the Ran/TC4 protein disrupts nuclear function in Xenopus laevis egg extracts by inhibiting the RCC1 protein, a regulator of chromosome condensation. EMBO J..

[bib10] Deng M., Suraneni P., Schultz R.M., Li R. (2007). The Ran GTPase mediates chromatin signaling to control cortical polarity during polar body extrusion in mouse oocytes. Dev. Cell.

[bib11] Deng M., Li R. (2009). Sperm chromatin-induced ectopic polar body extrusion in mouse eggs after ICSI and delayed egg activation. PloS One.

[bib12] Duncan F.E., Moss S.B., Schultz R.M., Williams C.J. (2005). Par-3 defines a central subdomain of the cortical actin cap in mouse eggs. Dev. Biol..

[bib13] Dumont J., Petri S., Pellegrin F., Terret M.E., Bohnsack M.T., Rassinier P., Georget V., Kalab P., Gruss O.J., Verlhac M.H. (2007). A centriole- and RanGTP-independent spindle assembly pathway in meiosis I of vertebrate oocytes. J. Cell Biol..

[bib14] Halet G., Tunwell R., Balla T., Swann K., Carroll J. (2002). The dynamics of plasma membrane PtdIns(4,5)P_2_ at fertilization of mouse eggs. J. Cell Sci..

[bib15] Halet G., Carroll J. (2007). Rac activity is polarized and regulates meiotic spindle stability and anchoring in mammalian oocytes. Dev. Cell.

[bib16] Heasman S.J., Ridley A.J. (2008). Mammalian Rho GTPases: new insights into their functions from in vivo studies. Nat. Rev. Mol. Cell Biol..

[bib17] Holt J.E., Jones K.T. (2009). Control of homologous chromosome division in the mammalian oocyte. Mol. Hum. Reprod..

[bib18] Jégou A., Pincet F., Perez E., Wolf J.P., Ziyyat A., Gourier C. (2008). Mapping mouse gamete interaction forces reveal several oocyte membrane regions with different mechanical and adhesive properties. Langmuir.

[bib19] Kim S.H., Li Z., Sacks D.B. (2000). E-cadherin-mediated cell–cell attachment activates Cdc42. J. Biol. Chem..

[bib20] Larson S.M., Lee H.J., Hung P.H., Matthews L.M., Robinson D.N., Evans J.P. (2010). Cortical mechanics and meiosis II completion in mammalian oocytes are mediated by myosin-II and Ezrin–Radixin–Moesin (ERM) proteins. Mol. Biol. Cell.

[bib21] Li H., Guo F., Rubinstein B., Li R. (2008). Actin-driven chromosomal motility leads to symmetry breaking in mammalian meiotic oocytes. Nat. Cell Biol..

[bib22] Longo F.J., Chen D.Y. (1985). Development of cortical polarity in mouse eggs: involvement of the meiotic apparatus. Dev. Biol..

[bib23] Ma C., Benink H.A., Cheng D., Montplaisir V., Wang L., Xi Y., Zheng P.P., Bement W.M., Liu X.J. (2006). Cdc42 activation couples spindle positioning to first polar body formation in oocyte maturation. Curr. Biol..

[bib24] Maro B., Johnson M.H., Webb M., Flach G. (1986). Mechanism of polar body formation in the mouse oocyte: an interaction between the chromosomes, the cytoskeleton and the plasma membrane. J. Embryol. Exp. Morphol..

[bib25] Miki H., Miura K., Takenawa T. (1996). N-WASP, a novel actin-depolymerizing protein, regulates the cortical cytoskeletal rearrangement in a PIP2-dependent manner downstream of tyrosine kinases. EMBO J..

[bib26] Na J., Zernicka-Goetz M. (2006). Asymmetric positioning and organization of the meiotic spindle of mouse oocytes requires Cdc42 function. Curr. Biol..

[bib27] Oceguera-Yanez F., Kimura K., Yasuda S., Higashida C., Kitamura T., Hiraoka Y., Haraguchi T., Narumiya S. (2005). Ect2 and MgcRacGAP regulate the activation and function of Cdc42 in mitosis. J. Cell Biol..

[bib28] Padrick S.B., Rosen M.K. (2010). Physical mechanisms of signal integration by WASP family proteins. Annu. Rev. Biochem..

[bib29] Perry A.C., Verlhac M.H. (2008). Second meiotic arrest and exit in frogs and mice. EMBO Rep..

[bib30] Pfender S., Kuznetsov V., Pleiser S., Kerkhoff E., Schuh M. (2011). Spire-type actin nucleators cooperate with Formin-2 to drive asymmetric oocyte division. Curr. Biol..

[bib31] Pollard T.D., Borisy G.G. (2003). Cellular motility driven by assembly and disassembly of actin filaments. Cell.

[bib32] Prehoda K.E., Scott J.A., Mullins R.D., Lim W.A. (2000). Integration of multiple signals through cooperative regulation of the N-WASP–Arp2/3 complex. Science.

[bib33] Rogers N.T., Halet G., Piao Y., Carroll J., Ko M.S., Swann K. (2006). The absence of a Ca^2+^ signal during mouse egg activation can affect parthenogenetic preimplantation development, gene expression patterns, and blastocyst quality. Reproduction.

[bib34] Rohatgi R., Ma L., Miki H., Lopez M., Kirchhausen T., Takenawa T., Kirschner M.W. (1999). The interaction between N-WASP and the Arp2/3 complex links Cdc42-dependent signals to actin assembly. Cell.

[bib35] Rohatgi R., Ho H.Y., Kirschner M.W. (2000). Mechanism of N-WASP activation by Cdc42 and phosphatidylinositol 4,5-bisphosphate. J. Cell Biol..

[bib36] Sardet C., Speksnijder J., Terasaki M., Chang P. (1992). Polarity of the ascidian egg cortex before fertilization. Development.

[bib37] Schuh M., Ellenberg J. (2008). A new model for asymmetric spindle positioning in mouse oocytes. Curr. Biol..

[bib38] Simerly C., Nowak G., de Lanerolle P., Schatten G. (1998). Differential expression and functions of cortical myosin IIA and IIB isotypes during meiotic maturation, fertilization, and mitosis in mouse oocytes and embryos. Mol. Biol. Cell.

[bib39] Sun S.C., Wang Z.B., Xu Y.N., Lee S.E., Cui X.S., Kim N.H. (2011). Arp2/3 complex regulates asymmetric division and cytokinesis in mouse oocytes. PLoS One.

[bib40] Symons M., Derry J.M.J., Karlak B., Jiang S., Lemahieu V., McCormick F., Francke U., Abo A. (1996). Wiskott–Aldrich syndrome protein, a novel effector for the GTPase Cdc42Hs, is implicated in actin polymerization. Cell.

[bib41] Verlhac M.H., Lefebvre C., Guillaud P., Rassinier P., Maro B. (2000). Asymmetric division in mouse oocytes: with or without Mos. Curr. Biol..

[bib42] Verlhac M.H. (2011). Spindle positioning: going against the actin flow. Nat. Cell Biol..

[bib43] Vinot S., Le T., Maro B., Louvet-Vallée S. (2004). Two Par6 proteins become asymmetrically localized during establishment of polarity in mouse oocytes. Curr. Biol..

[bib44] Yasuda S., Oceguera-Yanez F., Kato T., Okamoto M., Yonemura S., Terada Y., Ishizaki T., Narumiya S. (2004). Cdc42 and mDia3 regulate microtubule attachment to kinetochores. Nature.

[bib45] Yi K., Unruh J.R., Deng M., Slaughter B.D., Rubinstein B., Li R. (2011). Dynamic maintenance of asymmetric meiotic spindle position through Arp2/3-complex-driven cytoplasmic streaming in mouse oocytes. Nat. Cell Biol..

[bib46] Zhang X., Ma C., Miller A.L., Katbi H.A., Bement W.M., Liu X.J. (2008). Polar body emission requires a RhoA contractile ring and Cdc42-mediated membrane protrusion. Dev. Cell.

